# Dynamic UAV Phenotyping for Rice Disease Resistance Analysis Based on Multisource Data

**DOI:** 10.34133/plantphenomics.0019

**Published:** 2023-01-16

**Authors:** Xiulin Bai, Hui Fang, Yong He, Jinnuo Zhang, Mingzhu Tao, Qingguan Wu, Guofeng Yang, Yuzhen Wei, Yu Tang, Lie Tang, Binggan Lou, Shuiguang Deng, Yong Yang, Xuping Feng

**Affiliations:** ^1^ College of Biosystems Engineering and Food Science, Zhejiang University, Hangzhou 310058, China.; ^2^ Huzhou Institute of Zhejiang University, Huzhou 313000, China.; ^3^ School of Information Engineering, Huzhou University, Huzhou 313000, China.; ^4^ Academy of Interdisciplinary Studies, Guangdong Polytechnic Normal University, Guangzhou 510665, China.; ^5^ Department of Agricultural and Biosystems Engineering, Iowa State University, Ames, IA 50011-3270, USA.; ^6^ College of Agriculture and Biotechnology, Zhejiang University, Hangzhou 310058, China.; ^7^ College of Computer Science and Technology, Zhejiang University, Hangzhou 310058, China.; ^8^ State Key Laboratory for Managing Biotic and Chemical Treats to the Quality and Safety of Agro-Products, Key Laboratory of Biotechnology for Plant Protection, Ministry of Agriculture, and Rural Affairs, Zhejiang Provincial Key Laboratory of Biotechnology for Plant Protection, Institute of Virology and Biotechnology, Zhejiang Academy of Agricultural Science, Hangzhou 31002, China.

## Abstract

Bacterial blight poses a threat to rice production and food security, which can be controlled through large-scale breeding efforts toward resistant cultivars. Unmanned aerial vehicle (UAV) remote sensing provides an alternative means for the infield phenotype evaluation of crop disease resistance to relatively time-consuming and laborious traditional methods. However, the quality of data acquired by UAV can be affected by several factors such as weather, crop growth period, and geographical location, which can limit their utility for the detection of crop disease and resistant phenotypes. Therefore, a more effective use of UAV data for crop disease phenotype analysis is required. In this paper, we used time series UAV remote sensing data together with accumulated temperature data to train the rice bacterial blight severity evaluation model. The best results obtained with the predictive model showed an *R*_p_^2^ of 0.86 with an RMSE_p_ of 0.65. Moreover, model updating strategy was used to explore the scalability of the established model in different geographical locations. Twenty percent of transferred data for model training was useful for the evaluation of disease severity over different sites. In addition, the method for phenotypic analysis of rice disease we built here was combined with quantitative trait loci (QTL) analysis to identify resistance QTL in genetic populations at different growth stages. Three new QTLs were identified, and QTLs identified at different growth stages were inconsistent. QTL analysis combined with UAV high-throughput phenotyping provides new ideas for accelerating disease resistance breeding.

## Introduction

Bacterial blight (BB) is a disease caused by *Xanthomonas oryzae* pv. *oryzae*. It has threatened the safety of rice production and brought irreversible damage to grains [1]. Currently, the main preventative measure against rice BB is the use of pesticides, which, in the long-term, could lead to pesticide resistance in the pathogen, as well as environmental pollution [2]. Planting disease-resistant cultivars is an economical and effective method to control crop diseases [3]. Marker-assisted selection has accelerated the process of crop breeding, which uses molecular markers closely linked to disease resistance genes to track and select target traits [4,5]. At present, the widely used molecular markers include simple sequence repeats (SSRs), sequence-tagged site (STS), and single-nucleotide polymorphis. Genetic. studies have shown that rice disease resistance is generally controlled by multiple major genes or the contributions from both major and minor genes [6]. Quantitative trait loci (QTL) mapping allows for the analysis of disease resistance. It can determine the genetic interactions between plant disease resistance and the function of specific resistance loci [7]. A large number of QTLs for BB resistance has been identified from different rice populations [8]. Some of these genes have been used to develop rice varieties resistant to BB, such as Xa3, Xa4, Xa7, Xa21, and Xa23 [9]. However, because of the continual emergence of new races of the pathogen, the resistance of the currently successful cultivars will eventually become less effective and even lost [10]. Exploring new resistance resources and applying them in disease resistance breeding is important. To verify the disease resistance, multiyear planting and phenotypic screening over many rice genotypes are needed [11]. The characteristics such as lesion length, lesion area, incidence rate, etc. are measured manually after inoculation [12–14]. Compared with BB-susceptible cultivars, lesions in resistant cultivars show reduced lengths and areas and occur in lower incidence [15]. The process of manual measurement is time-consuming, is laborious, and is affected by the individual’s subjective judgment. It is necessary to improve the efficiency and accuracy of disease severity analysis through the employment of effective high-throughput analytical methods.

In recent years, unmanned aerial vehicle (UAV) remote sensing has developed rapidly. With the advantages of flexibility, real-time data acquisition, and low cost, it provides a feasible way for the rapid acquisition of phenotypic information of crops in the field [16]. In practical application, UAV is equipped with image sensors (such as red–green–blue, hyperspectral, and multispectral cameras) to acquire the crop images. The changes of leaf/canopy morphological and structural changes, color and texture variation, biochemical component content changes, etc. caused by stress are reflected in the images [15]. These features can be extracted and quantified by image processing algorithms for use in the analysis of crop disease. Evolving machine learning methods have provided powerful tools for extracting and evaluating these features [17,18]. Sugiura et al. [19] evaluated the field resistance of potato late blight based on UAV time series of RGB images, and the coefficient of determination (*R*^2^) was 0.77. Su et al. [20] used time series 5-band (visible infrared) aerial imaging to identify sensitive spectral indicators of the severity of yellow rust disease in wheat through statistical correlation analysis. These earlier studies highlight the promise of UAV imaging and data processing methods for the assessment of disease severity in the field and the selection of disease-resistant cultivars.

With the application of UAV remote sensing technique in agriculture, the combination of high-throughput phenotypic data and genetic data can provide support to accelerate crop breeding. Wang et al. [21] reported that different genes were found to regulate plant height (PH) at different developmental stages, and mapping with dynamic phenotypic data could identify more QTLs, affecting the development of PH traits. Hassan et al. [22] discovered 2 new stable QTLs on chromosome 6 seemingly associated with accelerated plant growth at the booting stage using UAV-based data. In addition, compared with traditional manual measurement methods, UAV high-throughput phenotyping provided considerable QTLs for the study of biomass, yield, chlorophyll content, and stress resistance [23,24]. Therefore, the combination of UAV traits and QTL analysis is considered to provide a new idea for BB resistance breeding of rice.

The development of crop disease is affected by the dynamic interaction of host crops, pathogens, and environmental conditions [25]. The plant phenotype is also constantly changing during the different stages of growth. Previous studies have shown that phenotyping results can be improved by considering the crop growth period and environmental factors. For example, Li et al. [26] combined hyperspectral and meteorological data to improve the predictive stability of yield and grain protein content of winter wheat in different growing seasons; Li et al. [27] developed a crop biomass algorithm in wheat for all growth stages by integrating remote sensing data and phenology information from multiple regions, which showed good potential for the estimation of biomass at varying spatial–temporal scales in winter wheat. Accumulated temperature (AT) in crop cultivation is a measure of the sum of temperatures received over time (°C/day). It is believed that when a plant develops to a certain stage, the temperature it needs is relatively consistent [28]. AT can reflect the crop’s maturity, which is a good indicator of plant growth [29]. Therefore, AT data are introduced to be combined with UAV remote sensing data to evaluate the severity of BB.

In this paper, the disease severity of rice BB was evaluated on the basis of time series UAV remote sensing images. The aims were (a) to explore the feasibility of UAV remote sensing images combined with AT data to evaluate and predict the severity of rice BB in the field, (b) to study the scalability of the established evaluation method to different geographical sites, and (c) to combine the predicted disease severity with QTL analysis to find effective disease resistance genes.

## Materials and Methods

### Experimental design

**Fig. 1.**
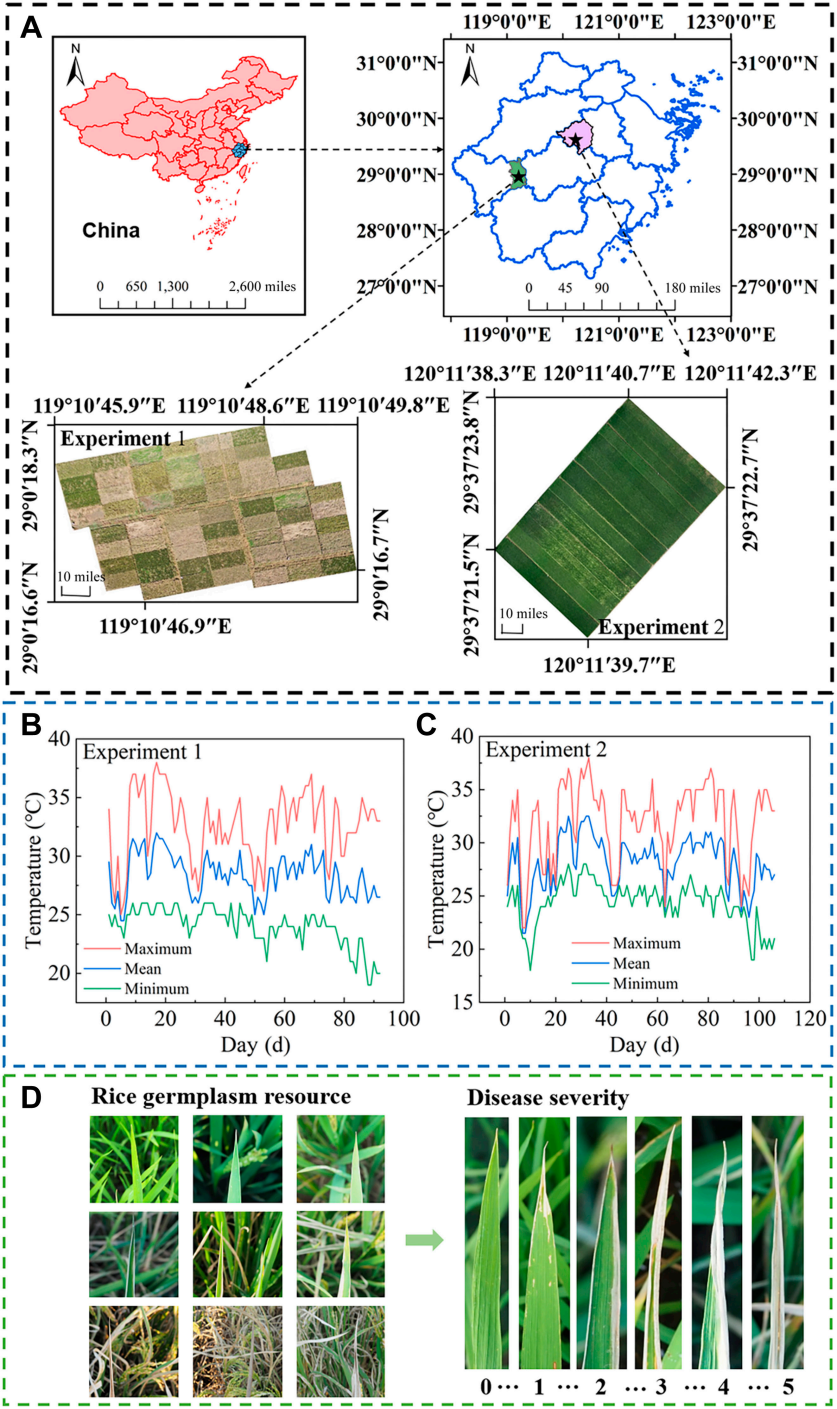
Geographical locations and related field data of study sites. (A) Location of the experimental sites 1 and 2. (B and C) The daily temperatures at sites 1 and 2, respectively. (D) Digital images of rice germplasm resources with different resistance to BB and quantifying the disease severity.

The data used in this study were collected from 2 experimental sites in 2021. Experiment 1 was conducted at Longyou County, Quzhou City, Zhejiang Province in China (Fig. 1A). It included 60 plots with an area of 10.60 m × 4.72 m for each plot, and 60 rice cultivars were planted. The 60 cultivars were obtained by constructing F8 generation recombinant inbred lines (RILs) from disease-resistant rice asymmetric somatic hybrid offspring (Bandaohong) and rice BB-sensitive IR24 as parents and were denoted as BI1-BI60. There were 5 low-resistant cultivars, 32 middle-resistant cultivars, and 23 resistant cultivars. Information on resistance was provided by the Zhejiang Academy of Agriculture Sciences. Rice seedlings were raised in mid-May 2021, transplanted manually to the field on June 26, and harvested in October. Experiment 2 was carried out at Paitou Town, Zhuji City, Zhejiang Province in China (Fig. 1A). The experimental field included 14 plots with 60 m × 5.50 m for each plot, and 13 rice cultivars with different resistance to BB were cultivated. The 13 cultivars planted were the main cultivars used in Zhejiang Province, and the resistance of each cultivar to BB had been determined by the National Rice Data Center (https://www.ricedata.cn/). There were 2 low-resistant cultivars and 11 middle-resistant cultivars. The rice seedlings were transplanted manually to the field on 13 June 2021, and the seedling raising time and harvest time were consistent with experiment 1. The interval between all adjacent plots in the 2 experimental fields was 0.50 m. Information about rice cultivars of the 2 experimental fields planted is detailed in Table S1. The climate of both locations belongs to the subtropical monsoon climate, with abundant rainfall and humid air suitable for rice growth. Importantly, these 2 experimental sites are endemic areas for BB, occurring naturally after rice planting without additional treatment. All the germplasm were obtained in 2020 and planted in 2021. The experimental materials were provided by the Zhejiang Academy of Agriculture Sciences.

The daily temperatures (minimum, maximum, and mean) were recorded at the 2 experimental sites from rice transplanting to the field (Fig. 1B and C). Then, AT data of each UAV campaign could be calculated according to the following formula:

$$\mathrm{AT}=\sum_{1}^{n}T_{\text{mean}}$$ (1)

where *n* is the number of days since transplanting, and *T*_mean_ is the daily mean temperature.

### Disease severity identification

The disease severity of rice BB in each plot was determined independently by 3 plant pathologists from the Zhejiang University and Zhejiang Academy of Agriculture Sciences according to the national standard GB/T 17980.19-2000 (http://std.samr.gov.cn/). Disease severity was quantified into 6 grades (as shown in Fig. 1D). Finally, the disease severity of the 3 pathologists was averaged as the disease severity of the plot (illustrated in Table S1).

### UAV image acquisition

UAV remote sensing images were acquired by a multirotor UAV developed by the Digital Agriculture and Agricultural Internet-of-things Innovation Laboratory at the Zhejiang University (Hangzhou City, Zhejiang Province, China), carrying a multispectral camera (MQ022MG-CM, XIMEA, Munster, Germany) and an RGB camera (NEX-7 camera, Sony, Tokyo, Japan) each mounted on a 3-axis gimbal in parallel to acquire images simultaneously (Fig. S1). The multispectral camera was equipped with a complementary metal-oxide semiconductor (CMOS) sensor (CMV2K CMOS, IMEC, Leuven, Belgium) and a 16-mm fixed lens (Edmund, Barrington, USA). It can acquire 23 wavelengths of a multispectral image with an image size of 409 × 216 pixels at the spectral region of 600 to 875 nm. The specific range of the wavelength bands are shown in Table S2. The spatial resolution of the RGB camera is 6000 × 4000 pixels. The flight control, information storage, and management of the UAV were controlled by the ground control station (CF-19 laptop, Panasonic, Kadoma, Osaka, Japan). An autopilot was attached to the UAV flight system to receive commands and flight plans from the ground station.

In 2021, flight campaigns were conducted 3 times in the 2 experimental fields. The weather was clear and cloudless, and the wind speed was low to reduce the image distortion affected by the weather condition. Data collection from experimental site 1 was undertaken on July 13 (early tillering stage), August 30 (jointing/heading stage), and September 25 (late filling stage), and experiment 2 was conducted on July 12 (tillering stage), August 19 (jointing stage), and September 26 (filling stage). The flight route was generated by Mission Planner (Mission Planner Software, San Diego, California, USA) of the ground control station. The camera equipped with UAV took pictures at the predefined flight route. Between l0:00 AM and 4:00 PM, the UAV was operated to acquire images at a flight altitude of 25 m and a speed of 2.5 m/s. The forward and lateral image overlaps were 60% and 75%, respectively. Before the UAV campaigns, a photometer (MQ-200, Apogee Instruments, Logan, UT, USA) was used to measure the light conditions to adjust the camera’s exposure time to prevent uneven images because of changes in light.

### Image preprocessing and feature extraction

Image mosaicking was conducted to obtain the orthophotos of the field’s multispectral and RGB images. A series of images were imported into the Agisoft PhotoScan Professional Software (Agisoft LLC, St. Petersburg, Russia). The orthophotos of the field could be exported through the steps of aligning images, building the dense cloud, constructing mesh, and producing orthomosaic. Georeferencing was performed using the set ground control points, and the geometric correction was performed using the affine transformation and nearest neighbor algorithm functions in MATLAB to eliminate image distortion. The orthophotos were recorded as digital number (DN) values, which should be converted to reflectance. In this study, a gray, 0.60-m × 1.2-m reference tarp of known reflectance (37%) was placed adjacent to the study area during each UAV campaign and used as a reference to convert the DN values to reflectance. The DN values of the tarp were extracted using ENVI 4.7 (ITT Visual Information Solutions, Boulder, Utah, USA). Then, the reflectance of each band image was calculated according to the formulas described by Wan et al. [30].

Furthermore, spectral correction was performed to remove unwanted optical responses, such as second-order responses and responses to filter crosstalk [31]. In the subsequent analysis, the spectral data used were the reflectance data in multispectral and RGB images, consisting of the 26 bands. Detailed information of 26 bands can be seen in Table S2. The extraction of the multispectral reflectance was consistent with the previous study [32]. The image at 675 nm with the maximum reflectance difference between the crop and background was selected to construct a mask for background segmentation by setting the threshold value as 0.1. The pixels of rice region were set as 1 and the background pixels were set as 0, and then the mask was applied to the grayscale image of each band to separate the rice from the background. A random forest classifier was used to remove the background for highlighting the rice canopy for RGB images [33]. During the data processing, the images of each plot in experimental site 1 were equally cut into 2 sub-images, and those from experimental site 2 were equally cut into 5 sub-images. Therefore, we had final image data of 106, 110, and 108 for experiment 1 and 70, 70, and 65 for experiment 2, respectively. Detailed information can be seen in Table S1.

The texture and color features of RGB images were extracted. According to the gray-level co-occurrence matrix, contrast, correlation, energy, and homogeneity were extracted from each band image. The detailed calculation can be found in [34]. The first, second, and third moments were calculated for color features as described in [35]. The RGB color space was also converted to hue–saturation–value (HSV) color space, and the conversion theory referred to [36]. The first moment, second moment, and third moment of the imaged in HSV color space were also calculated. These features were extracted in MATLAB R2019a (MathWorks, Natick, Boulder, CO, USA).

### Physiological parameters analysis

Physiological parameters of rice, including leaf chlorophyll content, fresh weight (FW), dry weight (DW), water content (WC), and leaf area index (LAI) in the field, were measured from 3 randomly selected rice plants from each plot. A plant canopy analyzer (LAI-2200C, LI-COR Inc., Lincoln, NE, USA) was used to measure the LAI values. Nondestructive measurement of chlorophyll was carried out using a SPAD-502 chlorophyll meter (Minolta Corp., Ramsey, NJ, USA). Subsequently, the aboveground part of the rice plant was harvested, weighed, and recorded as FW. Then, it was dried to a constant weight and recorded as DW. WC was calculated on the basis of the FW and DW [37].

### Statistical analysis

Pearson correlation was implemented to assess the correlation between the disease severity and spectral, AT, SPAD, WC, and LAI data of rice. Two-way analysis of variance (ANOVA) was applied to calculate differences in the physiological parameters and spectral data of rice between the 2 experimental fields among different disease severity [38]. Tukey’s post hoc method was used for multiple comparisons, and the values of *P* < 0.05 were considered statistically important [39]. The above statistical analysis was performed using SPSS Statistics 26.0 (IBM Corporation, Armonk, NY, USA) and Origin 2021 (OriginLab Corporation, Northampton, MA, USA).

Partial least squares regression (PLSR) and support vector regression (SVR) models were used to evaluate the severity of BB. PLSR conducts a regression analysis model based on linear transformation, while SVR establishes a regression model based on nonlinear transformation. Details of the algorithms can be found in [40,41]. A leave-one-out cross-validation approach was applied to train the PLSR model. The Gaussian radial basis function was selected as the kernel function, and grid search and 10-fold cross-validation were used to optimize the important parameters of the SVR model. The search range of penalty coefficient (*c*) and the kernel function parameter (gamma) were integers in the 10^−2^ to 10^10^ and 10^−9^ to 10^3^, respectively. The PLSR and SVR models were implemented using scikit-learn (https://scikit-learn.org/stable/), a machine learning tool kit for Python.

### Model updating strategy

Model updating is where the initial model created is updated with new data for model training, which can make it more robust to handle more variations in the field. This can be achieved while retaining the majority of the initial training set, therefore requiring relatively less modeling time between forays [42–44]. The scalability of the established model between different sites was investigated with model updating strategy. Since more cultivars were available at experimental site 1, we initially used the data from this site as the calibration set to establish SVR models for the prediction of BB disease severity in the dataset from experimental site 2. Then, the data from experimental site 2 were randomly selected to be added to the calibration set form site 1 at a ratio of 10% to 90% in 10% intervals. The remaining data of experimental site 2 were used as the prediction set to test the predictive performance of the updated model.

### Deep spectral features extraction

As a deep feedforward artificial neural network, convolutional neural network (CNN) has a powerful learning ability and has been proven to be an effective feature extraction method [45]. Compared with other methods, CNN can automatically extract features without human intervention [46]. In this paper, a simple CNN architecture was used to extract deep spectral features. The specific network structure and parameters utilized are shown in Fig. S2. It consisted of 2 convolution blocks, 2 dense layers, and an output layer. The number of kernels of the convolutional 1D was 16, with a kernel size of 3 and stride of 1. Exponential linear unit activation was used as the activation function. A maxpooling layer with a pool size of 2 and a stride of 2, and a batch normalization layer followed. The neurons of the 2 dense layers were 128 and 16, and the rectified linear unit was used as an activation function. Both of the dense layers were followed by a batch normalization layer. At the end, a dense layer with 1 neuron was added for output. The CNN architecture was built in MXNet1.4.0 (MXNetAmazon, Seattle, WA, USA).

As a feature extractor, the CNN model was trained for regression tasks and output the deep spectral features. The loss function was L2loss. In the training phase, the batch size was set as 5, and a dynamic learning rate was used. At the beginning, a relatively large learning rate of 0.0005 was set to speed up the training process for the first 300 epochs, and then it was reduced to 0.00005 for the next 300 epochs. The spectral data of 26 bands of each plot were input into the CNN model, and the SPAD/WC of rice was used as the dependent variable for the regression task. The output of the second batch normalization layer (16 deep spectra features) was extracted as deep spectral features, which were input into the SVR model to predict the disease severity.

### Model evaluation

The data were randomly divided into calibration set and prediction set at a ratio of 8:2 for the establishment of models. The *R*^2^, root mean square error (RMSE), and residual prediction deviation (RPD) were used to evaluate the model performance. The calculations utilized are described in [47]. *R*^2^_c_ and RMSE_c_ represented the calibration set results, and *R*_p_^2^ and RMSE_p_ represented the results of the prediction set. A good performance model has a value for *R*^2^ close to 1, with a lower RMSE and a higher RPD. RPD > 2.5 means that the model has excellent predictive ability, 2 < RPD < 2.5 means a good model, 1.5 < RPD < 2 means a relatively fair model, and RPD < 1.5 means that the model is incapable of prediction [48].

### Quantitative trait loci

QTL is the location in the genome where a gene controls quantitative traits [49]. Mapping crop responses under pathogen stress will help breeders identify functions or traits of crops that QTLs control. In this study, QTL mapping was conducted for disease severity obtained by analyzing UAV data from experimental site 1. DNA of the 60 RILs and parents were extracted using the method described in [50]. A total of 514 pairs of SSR and 101 pairs of STS were selected from the rice microsatellite website (http://www.gramene.org/microsat/) and National Center for Biotechnology Information (http://www.ncbi.nlm.nih.gov/), respectively, which were evenly distributed throughout the rice chromosomes. The polymorphisms of the primer pairs between the parents were analyzed, and the genetic linkage map of the polymorphic molecular markers was constructed using Windows QTL Cartographer V2.5 [51]. Kosambi’s mapping function was used to transform the recombination frequency into the genetic distance (cM). The map contained 155 SSR and 56 STS, covering about 1540.0 cM of the rice genome region with an average map distance of 7.89 cM. Compound interval mapping (CIM) was subsequently used to conduct QTL mapping for disease resistance based on the disease severity predicted from the UAV data. The genome scanning interval was 2 cM. Permutation analysis (1000 iterations) was used to establish experimental significance at a 0.05 confidence level for each trait in CIM, and default parameters of the software were used for other parameters. A logarithm of odds (LOD) value of 3 was used as the threshold to determine the existence of QTL. In addition, the estimated percentage of phenotypic variation explained (R2) was calculated to reflect the relative contribution of a locus to a trait, and the additive effect (A) of each QTL was determined. The disease severity predicted from UAV data included the predicted results after 3 UAV campaigns (denoted time 1, time 2, and time 3) during the period up to rice harvest. There were 60 cultivars in the first UAV campaign. However, because of rice lodging in the field, only 54 cultivars remained for the second and third campaign for use in QTL analysis.

## Results

### Relationship between physiological parameters of rice and BB severity

**Fig. 2.**
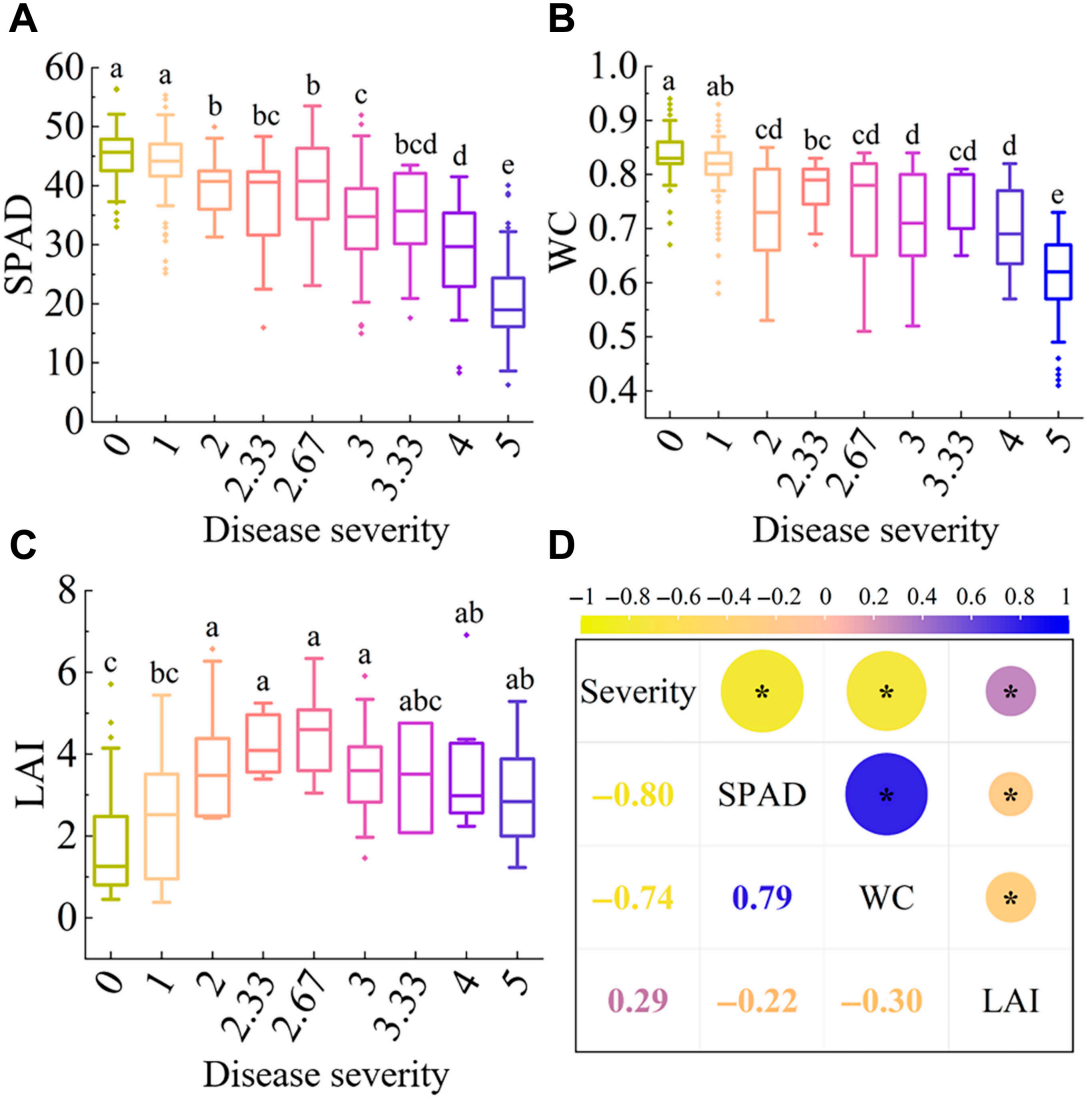
Box plots of SPAD (A), WC (B), and LAI (C) of rice with different disease severity. The data presented were taken from both experimental sites. Different letters on the top of boxes indicate significant differences (*P* < 0.05) in the physiological parameters at different levels of disease severity as determined by Tukey test. (D) Correlation coefficient among disease severity, SPAD, WC, and LAI (*p < 0.05).

The physiological parameters were changed under the stress of BB, as shown in Fig. S3. It can be observed that the variation trend of these parameters of rice between the 2 sites was basically consistent with the aggravation of disease severity. There are no significant differences in SPAD, WC, and LAI between different sites with different disease severity (Fig. S3A, B, and E). The changes of SPAD, WC, and LAI of rice under the stress of BB were unrelated to the geographical location and cultivars. Figure 2A to C shows the changes of SPAD, WC, and LAI in rice by pooling the field data from the 2 experimental sites among different disease severity. The values of SPAD and WC were both decreased with increasing disease severity, while the change of LAI was not as regular as SPAD and WC. Pearson’s correlation analysis was used to assess the correlation between the disease severity and SPAD, WC, and LAI of rice (Fig. 2D). The SPAD and WC of rice had robust negative correlations with the severity of BB with the values of *r* of −0.80 and −0.74, respectively.

Previous studies showed that it was feasible to use spectral data to evaluate the disease severity, chlorophyll content, and WC of rice [52,53]. The changes in rice chlorophyll content and WC were related to the development of rice BB, which led to differences in spectral data [9]. The correlations observed here between BB severity and SPAD and WC prompted us to investigate whether the spectral features of SPAD or WC could be used to evaluate disease severity.

The correlation analysis among each spectral band and disease severity was conducted on all data from the 2 experimental sites. As shown in Table S3-1, there were significant correlations among band 14 to band 25, which might cause information redundancy. The CNN network was used as a feature extractor to extract deep spectral features of SPAD and WC, and the prediction results of the CNN model are shown in Table S3-2. For SPAD, the *R*_p_^2^ was 0.79, the RMSE_p_ was 4.81, and the RPD was 2.19. The *R*_p_^2^ of WC was 0.79, with an RMSE_p_ and RPD of 0.04 and 2.17, respectively. The results indicated that the evaluation of SPAD and WC using spectral data was reliable. On the basis of these results, the output of the batch normalization layer followed the second dense layer of the CNN model that was extracted as deep spectral features and input into the SVR model to evaluate the disease severity. Promising results are obtained and shown in Fig. S4. For SPAD, the *R*_p_^2^ was 0.83, the RMSE_p_ was 0.70, and the RPD was 2.46. The *R*_p_^2^ of WC was 0.76, with an RMSE_p_ and RPD of 0.84 and 2.05, respectively. In comparison, the model performed on the deep spectral features extracted based on SPAD was relatively reliable. As a feature extractor, CNN has learned some important spectral information, which is important for evaluating disease severity. It was therefore considered feasible to indirectly evaluate the severity of rice BB from the evaluation of SPAD or WC.

### Disease severity evaluation based on UAV data fused with AT

**Fig. 3.**
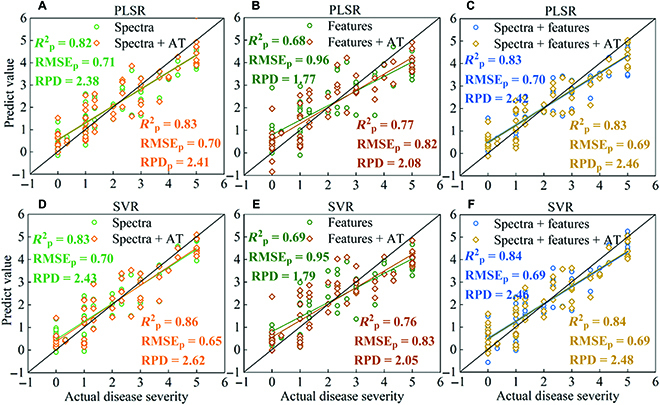
The predicted results of PLSR (A to C) and SVR (D to F) models based on different datasets. Spectra represent the use of spectral data. Features represent the use of texture and color; features + AT represent the added use of AT data.

The feasibility of adding AT data to the UAV data for the evaluation of BB severity was explored using the data acquired from experimental site 1. PLSR and SVR models with and without AT data were established for comparison. Figure 3 shows the prediction results, and Table S4 shows all the calibration and prediction set results.

As shown in Fig. 3A and D, using spectral data alone, the *R*_p_^2^ of the PLSR and SVR models exceeded 0.82, with an RPD above 2.3 indicating that the evaluation of disease severity using spectral data was reliable. After introducing AT data into spectral data for modeling, the PLSR and SVR models’ performances were slightly improved. For the PLSR model, the *R*_p_^2^ increased from 0.82 to 0.83, the RMSE_p_ decreased from 0.71 to 0.70, and the RPD increased from 2.38 to 2.41. For the SVR model, the *R*_p_^2^ increased from 0.83 to 0.86, the RMSE_p_ decreased from 0.70 to 0.65, and the RPD increased from 2.43 to 2.62. The inclusion of AT data therefore improved the prediction of BB disease severity.

Texture and color features of RGB images were also used to evaluate the disease severity of rice BB. First, an analysis of the correlation between these features and disease severity showed several with no significant correlation (Table S5). Therefore, the features with an absolute correlation coefficient value greater than 0.7 were selected. The results of the PLSR and SVR models are shown in Table S4 and Fig. 3B and E. The RPD of the 2 models was less than 2, which proved that using the features data for evaluating disease severity needs to be deliberated. However, the performance of PLSR and SVR models was significantly improved when the AT data were considered. The *R*_p_^2^ increased by 13.24% and 10.14%, the RMSE_p_ decreased by 14.58% and 12.63%, and the RPD increased by 14.90% and 14.53% (Fig. 3B and E). The RPDs of the 2 models were both over 2, indicating that the introduction of AT data for evaluating disease severity was considerable.

The case of combining spectral data with texture and color features for evaluating disease severity was also considered. As shown in Table S4 and Fig. 3C and F, the results of the PLSR and SVR models were slightly improved compared with the results of using spectral data alone. The *R*_p_^2^ of both models increased by 0.01, and RMSE_p_ decreased by 0.01. After the introduction of AT data, the performances of the PLSR and SVR models were unchanged. The results showed that the spectral information of the remote sensing images was relatively important for the disease severity evaluation of rice BB, compared to features data. The fusion of spectral data and AT could obtain stable experimental results.

Regarding model selection, the results of SVR models were slightly better than PLSR models. Especially for evaluating disease severity based on spectral data, the SVR model of spectral data combined with AT data for the prediction of BB severity achieved an optimal result with an RPD of 2.62, which can be regarded as an excellent predictive ability (Fig. 3D). Therefore, only the spectral data were considered used for the disease severity evaluation of rice BB in the subsequent analysis.

### Model updating for evaluating disease severity over different experimental sites

**Fig. 4.**
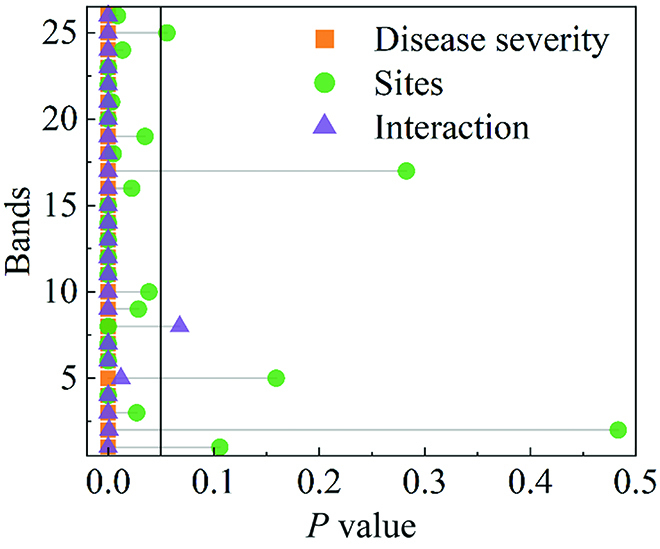
Two-way ANOVA result of the 2 experimental sites under the same disease severity in each band (disease severity represents the ANOVA for different disease severity, sites represents the ANOVA for the 2 experimental sites, interaction represents the 2-way interactions, the black line indicates the threshold *P* value of 0.05, and the corresponding wavelengths of bands are detailed in Table S2).

To establish a relatively universal model for disease evaluation, the data of the 2 experimental sites were analyzed comprehensively. The spectral profiles with different disease severity of experimental site 1 are shown in Fig. S5. In the visible region, spectral reflectance increased with disease severity, whereas in the near-infrared region, spectral reflectance decreased with disease severity. Similar trends in reflectance with BB disease severity were found in the spectral data from experimental site 2 (Fig. S6). The variation trend of reflectance with the aggravation of BB was consistent between the 2 sites, but the reflectance values differed. The *P* values of the interaction term were less than 0.05 except for the band 8, which indicated that the spectral data of different disease severity were different between different sites (Fig. 4 and Table S6). The effects of different severity and different sites on spectral reflectance were significant. Next, the model’s scalability for evaluating the rice BB severity between 2 experimental sites was explored. The spectral data of the 2 sites and AT data were used to establish the SVR model for analysis.

**Fig. 5.**
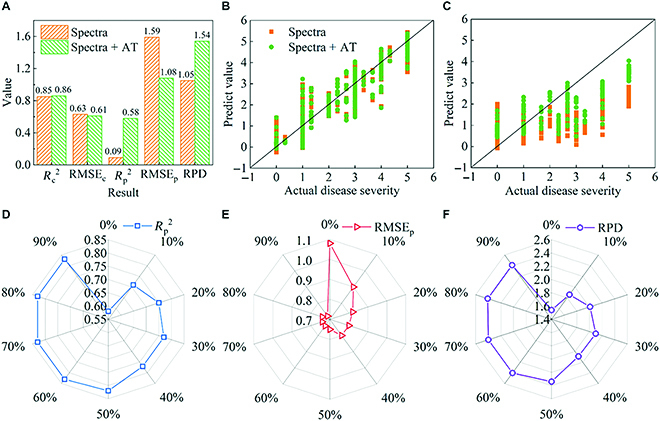
Results of model updating. (A) The *R*^2^, RMSE, and RPD achieved when data from experimental site 1 were used as the calibration set and the data of experimental site 2 as the prediction set. (B and C) The predicted and actual values of the calibration set and prediction set (the black lines are the 1:1 line). The *R*_p_^2^ (D), RMSE_p_ (E), and RPD (F) obtained from SVR models established with different ratios of the transferred data from experimental site 2 to experimental site 1.

Figure 5A to C displays the results of taking the spectral data from experimental site 1 as the calibration set and that from experiment site 2 as the prediction set to evaluate the predictive capability of the initial model. As expected, it can be seen that, when used against the calibration set from site 1, the predicted values of disease severity by the model showed a good correlation with actual values. However, the predicted values from the model for experimental site 2 were generally lower than the actual disease severity (Fig. 5C), with an RPD of less than 2, indicating an unreliable predictive capability when used against geographical sites not considered in the training data (Fig. 5A and Table S7-1). However, the prediction effect of the model was significantly improved by introducing the AT data. The *R*_p_^2^ was 0.09 using only spectral data for modeling, but reached 0.58 when in combination with AT data. The RMSE_c_ and RMSE_p_ both showed a decreasing trend, while RPD showed an increasing trend with the introduction of AT data. The RMSE_p_ decreased by 32.08%, and the RPD increased by 46.67%. The results indicated that the introduction of AT data can significantly improve the reliability of the predicted of disease severity across different sites. Therefore, the spectral data combined with AT data were used for subsequent analysis.

To improve the scalability of the model to different geographical sites, model updating with differing levels of data from experimental site 2 (0% to 90%) was transferred to form new training sets, and the predictive performance of the resulting models tested against the remaining data from experimental site 2. The results are shown in Fig. 5D to F and Table S7-2.

The *R*^2^ and RMSE obtained with the models against the training data fluctuated relatively slightly with the increasing ratio of transferred data. However, when used against the prediction set, the model displayed substantially improved performance, with increases in *R*_p_^2^ and decreases in RMSE_p_. Although greater performance was consistently achieved with higher ratios of transfer, the rate of improvement was highest over the 0% to 50% transfer range and was reduced thereafter (Fig. 5D to F). With a transfer of 20%, the updated model achieved a good predictive capability, with an increase in the RPD from 1.54 to 2.01. Finally, the spectral data of the 2 sites were pooled to evaluate the disease severity. The SVR model was established by using spectral data fused with AT data. Promising performance was acquired (*R*_p_^2^ = 0.85, RMSE_p_ = 0.65) (Fig. S7).

### Identification of QTLs

**Fig. 6.**
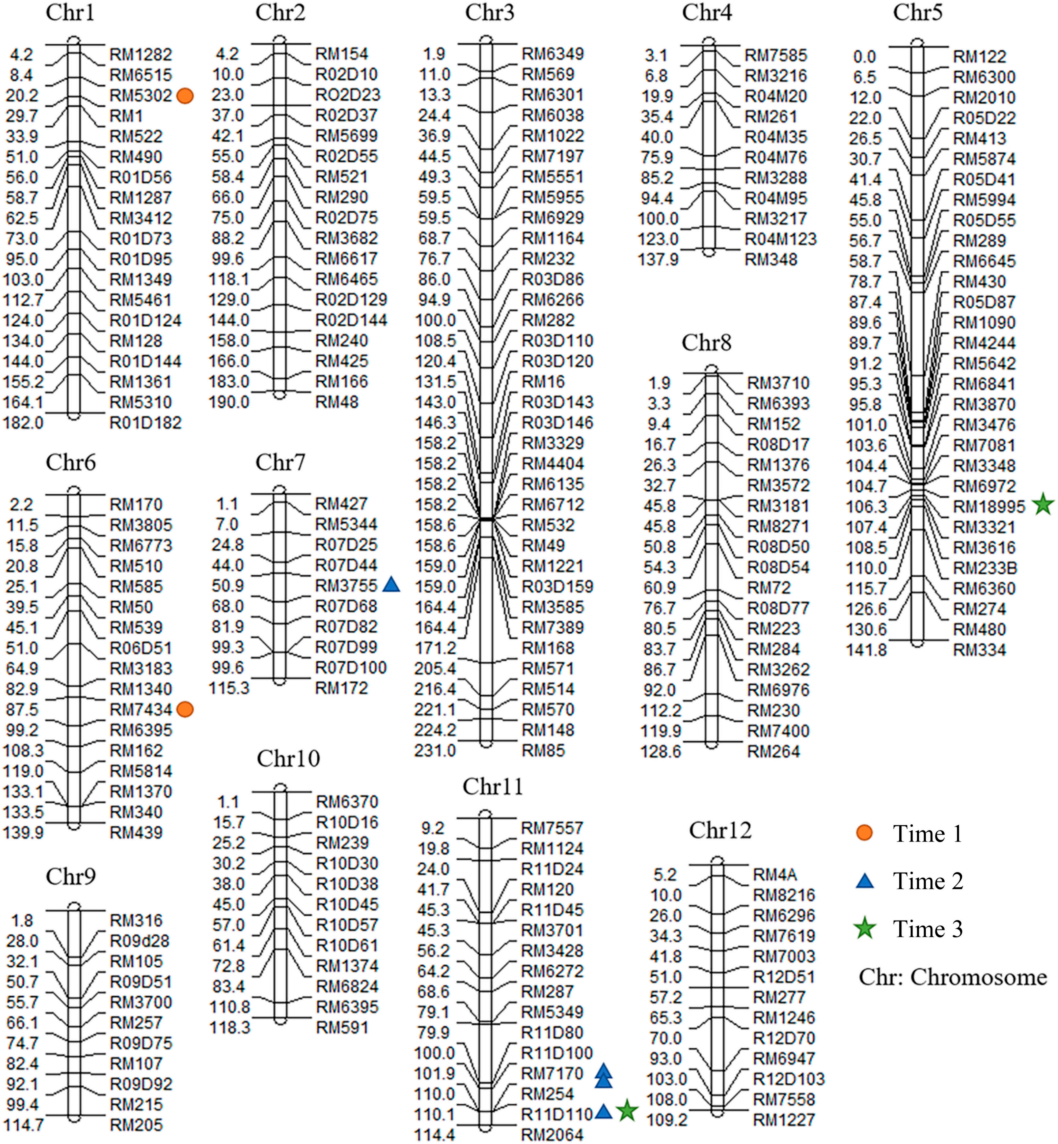
The linkage map of QTLs detected in the genetic population. The genetic distance (cM) between adjacent markers and marker names is displayed on the chromosomes’ left and right sides, respectively. Symbols of different shapes and colors on the right side of the chromosome indicate the location of the detected QTL (time 1, time 2, and time 3 represent QTL mapping using the predicted disease severity results of rice after UAV campaigns as phenotypic traits).

The results of QTL mapping are shown in Fig. 6 and Table. A total of 8 resistance QTLs were detected, and the QTLs detected in different periods were different. The 8 QTLs were distributed on chromosomes 1, 5, 6, 7, and 11, of which 4 QTLs were located on chromosome 11. Each QTL explained the phenotypic variation from 14% to 35%. Seven QTLs had positive additive effect values, indicating that synergistic alleles at these loci derived from the resistant parents, whereas 1 QTL, qBB1R6 on chromosome 6, had a negative additive effect.

**Table.** QTLs for rice bacterial blight resistance detected in rice at different growth stages. Time 1, 2, and 3 represent QTL mapping using the predicted disease severity results of rice after 3 UAV campaigns as phenotypic traits.

Time	QTL	Chr	Market interval	Near marker	LOD	R2, %	A
1	qBB1R1	1	RM6515–RM522	RM5302	6.04	35	0.17
	qBB1R6	6	RM1340–RM6395	RM7434	4.01	19	−0.15
2	qBB2R7	7	R07D44–R07D68	RM3755	3.38	14	0.37
	qBB2R11a	11	R11D100–RM254	RM7170	3.96	19	0.38
	qBB2R11b	11	R11D100–RM254	RM7170	4.01	23	0.46
	qBB2R11c	11	RM254–RM2064	R11D110	5.77	22	0.5
3	qBB3R5	5	RM6972–RM3321	RM18995	5.48	22	0.52
	qBB3R11	11	RM254–RM2064	R11D110	3.83	15	0.5

QTL, quantitative trait locus; Chr, chromosome; R2, the estimated percentage of phenotypic variation explained; A, additive effect.

Of the 7 QTLs with positive additive effects, 4 were located on chromosome 11. Three of the 4 QTLs (qBB2R11a, qBB2R11b, and qBB2R11c) were detected in time 2 and 1 (qBB2R11) in time 1. The QTLs qBB2R11c and qBB2R11 were both located in the RM254-RM2064 interval, adjacent to R11D110 and close to 110.3 cM on the genetic map.

## Discussion

### The combination of UAV data and AT data can improve the evaluation effect of rice BB severity

Previous studies have demonstrated that it is feasible to use UAV equipped with optical imaging for crop disease detection [20,54]. The spectral information, texture features, color features, etc. of optical images are important information to reflect crop growth and disease distribution in the field. However, there are relative few studies on rice BB. In this paper, UAV campaigns were conducted 3 times at the 2 experimental sites to explore the feasibility of evaluating the severity of BB from UAV data.

The development of rice BB disease in the field is a dynamic process, with an increase in the display of symptoms (lesions, leaf yellowing, and plant withering) as the disease progresses in severity, and this progression is affected by the plant stage of development [55,56]. For many researches, data acquisition and analysis were almost performed on crops in the same stage, and the results of disease evaluation models were different under different growth stages. In the current research, the developmental stages of rice covered by the 3 UAV campaigns extended from early tillering to the late filling stage, which can affect the functional deployment of resistance genes. AT is an important index for crop growth and development. In agricultural modeling applications, AT data can be used to accurately predict the rate of plant growth and development, thereby reducing the influence of temperature variations and improving the models predictive performance [29]. Different rice cultivars have different resistance to BB, and the disease severity of rice may be different at different growth stages. In agreement, we observed that the combination of spectral and AT data showed an improved prediction of BB disease severity (Fig. 5). In addition, when analyzing the data between the 2 experimental sites, it was found that the applicability of the model to the different sites was significantly improved after the introduction of the AT data (Fig. 5A). When using the model updating strategy, the introduction of AT data was also seen to improve the evaluation of rice BB severity (Tables S7-2 and S7-3).

### Model updating strategy facilitates the evaluation of BB severity over different sites

The initial model was developed from training data from experimental site 1 alone and showed only a fair-good predictive capability of BB severity against new data obtained from experimental site 2. A comparative analysis of the UAV data from the 2 experimental sites showed significant differences in reflectance values obtained at different spectral bands (Fig. 4). The use of a model updating strategy has been reported to effectively improve the predictive power against different datasets [44]. In this study, the model’s performance was significantly improved by transferring 20% of the new data to the training dataset. As the proportion of the new transferred data exceeded 50%, the improvement tended toward maximal values, indicating that the majority of useful information had been assimilated. Considering the cost of field sampling, a transfer of 20% of new data was considered a useful and cost-effective model update strategy to achieve reliable predictions of BB severity across different sites.

### New resistance QTLs have been detected based on dynamic traits

At present, many QTLs related to BB resistance have been detected, and these QTLs are distributed on 12 chromosomes of rice [57]. This study detected both previously reported and novel QTLs for resistance. For example, qBB2R7 overlapped with the QTL marker intervals reported by Vikal et al. [58], but the nearest marker interval differed. The qBB2R11a, qBB2R11b, qBB2R11c, and qBB2R11 were located at the end of the long arm of chromosome 11, which is a region enriched in BB resistance genes, including Xa4, Xa30, Xa32, Xa35, and Xa36 [8]. Three new QTLs related with BB resistance were identified. The three new QTLs shows that the resistance parent has new BB resistance genes, and the resistance genes are inherited. QTL analysis combined with UAV high-throughput phenotyping provides new ideas for disease resistance breeding.

The identification of resistance genes functional at all stages of development can be substantially useful in resistance breeding programs. However, it is known that more resistance genes are expressed in the adult stage of rice development compared with the seedling stage [59]. In this paper, the identification of QTLs from phenotyping data revealed that none was evident at all the 3 stages of development assayed, indicating that BB resistance genes that show continual expression throughout this developmental period are rare. Of the QTLs, qBB1R1 has a positive additive effect in resistance with a phenotypic contribution of 35% in the early tillering stage (time 1) but was not detected in later stages. Similarly, 3 QTLs were detected only at the jointing/heading period of development (time 2). An identical QTL was detected in these 2 stages, with the adjacent marker R11D110. It indicates genetic overlap in the quantitative genetic mechanism of BB resistance from jointing to maturity stage of rice. In addition, at time 2, 4 QTLs were detected. At this stage, rice was mostly at the jointing and heading stage, at which BB often outbreak [1]. The expression of the resistance gene was significant. There are spatial–temporal expression phenomena of BB resistance genes.

### Conclusion

This study examined the feasibility of using aerial spectral data from rice paddy fields to construct an effective model for the prediction of BB disease severity and, consequently, to analyze the phenotypic variations shown by a RIL population of differing resistances. The combination of spectral and AT data was found to improve the reliability of the predicted BB disease severity. A model updating strategy was employed to achieve the scalability of the model to different geographical sites of rice cultivation. In addition, 3 new QTLs were identified by combining QTL analysis with UAV remote sensing. Genetic overlap exists in the variation of resistance information at different growth stages. UAV remote sensing is a reliable method for mining of BB resistance genes. It is of great significance for the genetic basis analysis of disease resistance and the improvement of traits. Compared with manual measurement of disease severity, UAV remote sensing technique can obtain large-scale phenotypic information rapidly, which provides technical support for accelerating breeding research. Although our model was developed specifically for rice BB, our overall strategy could be extended to other crops and their diseases to achieve a wider application.

## Data Availability

The data supporting the findings of this study are available from the corresponding author (X.F.) upon request.

## Supplementary Materials

Supplementary 1 Table S1. Information about the 2 experiments. Table S2. The corresponding wavelengths of the 26 bands of spectral data used in this study. Table S3. The confusion matrix of correlation coefficient among disease severity and each spectral band, AT, SPAD, and WC. Table S4. The results of PLSR and SVR models for the disease severity evaluation of rice bacterial disease based on different datasets. Table S5. The confusion matrix of the correlation coefficients between disease severity and texture and color features of experiment 1. Table S6. The ANOVA table from the 2-way ANOVA considering the disease severity and sites with the interaction term. Table S7. The results model updating.

Supplementary 2 Fig. S1. The UAV platform. Fig. S2. The architecture of CNN. Fig. S3. Box plots of physiological parameters of rice with different disease severity in the 2 experimental sites. Fig. S4. The results of SVR model established by using the deep spectral features extracted by CNN based on SPAD and WC of rice. Fig. S5. Averaged spectral curves of different disease severity in experiment 1. Fig. S6. Averaged spectral curves of different disease severity in experiment 2. Fig. S7. The results of SVR model established for the evaluation of disease severity by using spectral data fused with AT data.
