# Supplementary material for: Dynamic UAV Phenotyping for Rice Disease Resistance Analysis Based on Multisource Data
Source: Plant Phenomics. 2023 Jan 16;5:0019. doi: 10.34133/plantphenomics.0019 (PMC10076055; doi:10.34133/plantphenomics.0019)
Supplement: Supplementary 2 — Fig. S1. The UAV platform. Fig. S2. The architecture of CNN. Fig. S3. Box plots of physiological parameters of rice with different disease severity in the 2 experimental sites. Fig. S4. The results of SVR model established by using the deep spectral features extracted by CNN based on SPAD and WC of rice. Fig. S5. Averaged spectral curves of different disease severity in experiment 1. Fig. S6. Averaged spectral curves of different disease severity in experiment 2. Fig. S7. The results of SVR model established for the evaluation of disease severity by using spectral data fused with AT data. [file plantphenomics.0019.f2.docx]

**Supplementary Figures:**


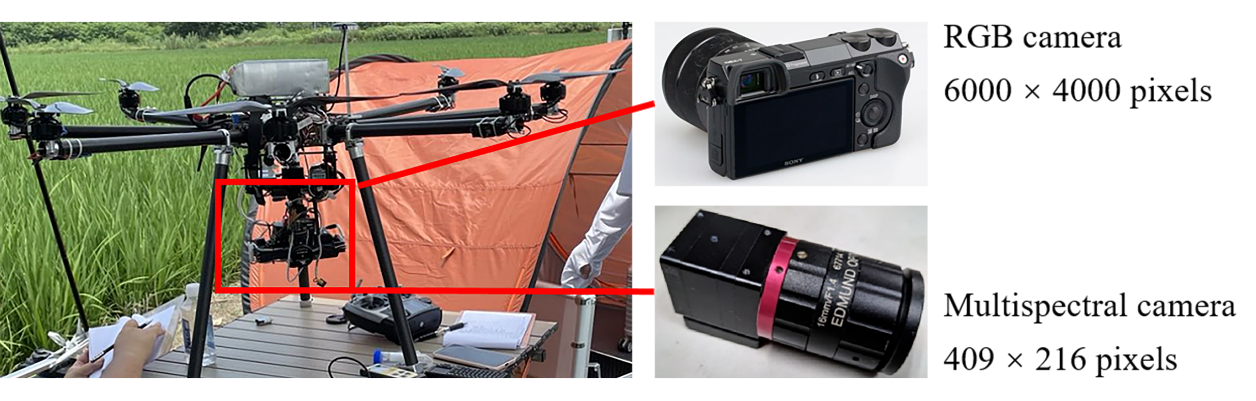


**Figure S1. The UAV platform.**

| 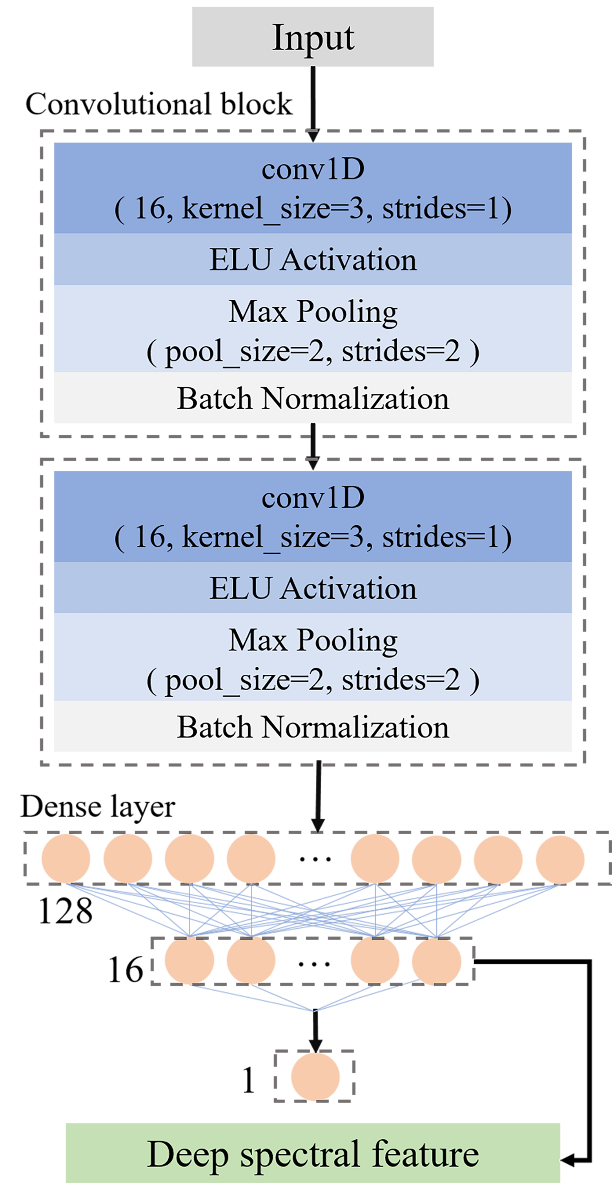 |
| --- |

**Figure S2. The architecture of CNN.**

| **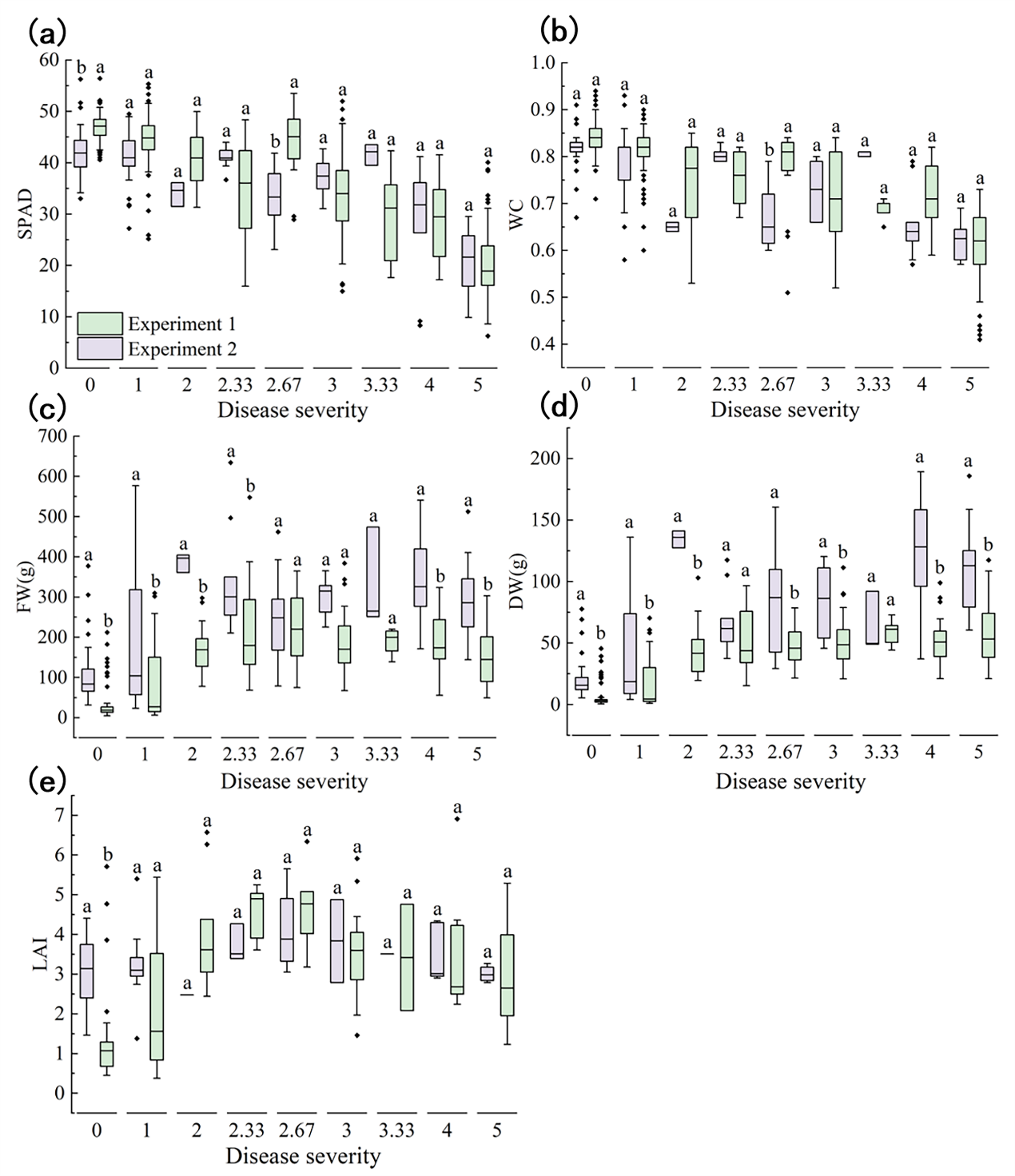** |
| --- |

**Figure S3. Box plots of (a) SPAD value, (b) water content (WC), (c) fresh weight (FW), (d) dry weight (DW), and (e) LAI of rice under different disease severity in the two experimental fields. Different letters on the top of boxes indicate significant differences between the two sites after ANOVA (P<0.05).**

| 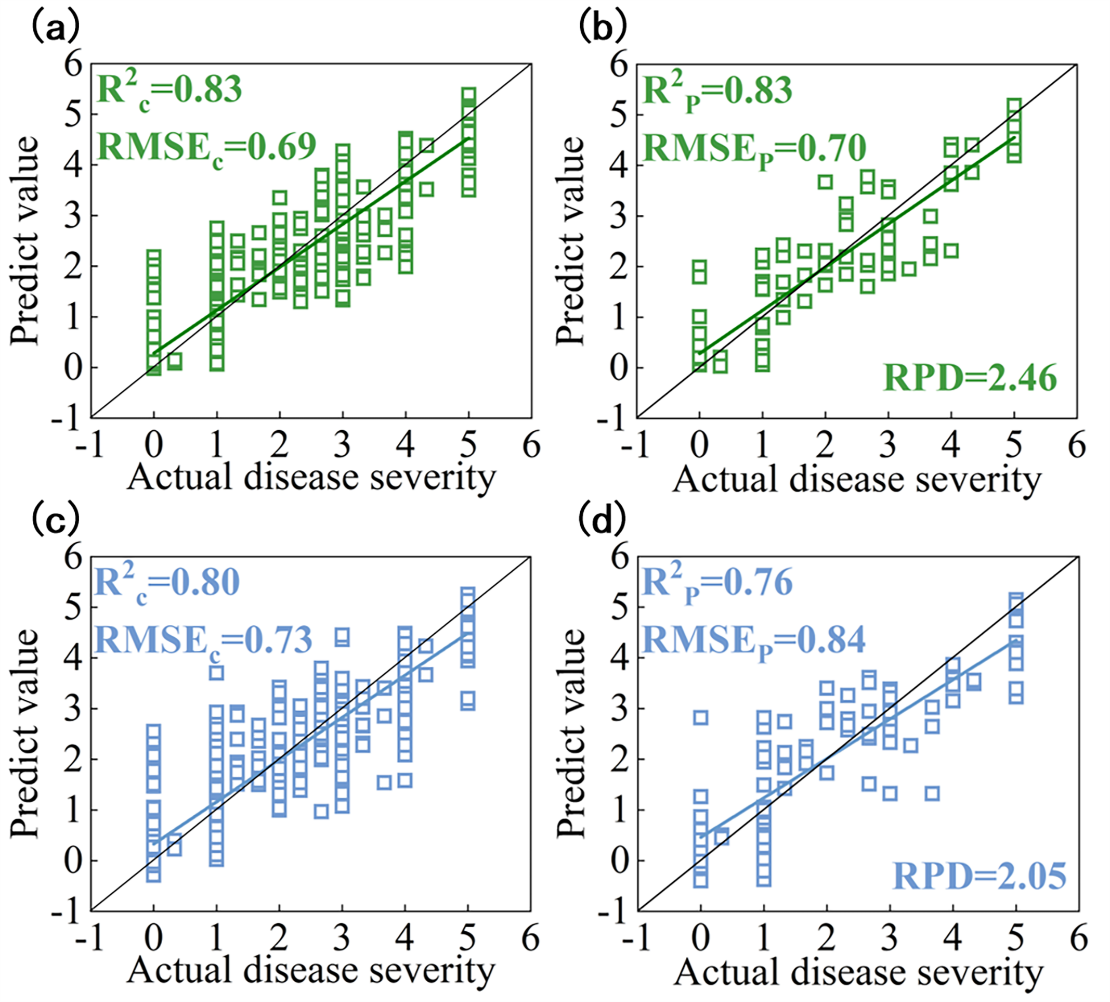 |
| --- |

**Figure S4. The results of calibration set (A) and prediction set (B) of the SVR model established by using the deep spectral features extracted by CNN based on SPAD of rice; The results of calibration set (C) and prediction set (D) of SVR model established by using the deep spectral features extracted by CNN based on WC of rice.**

| **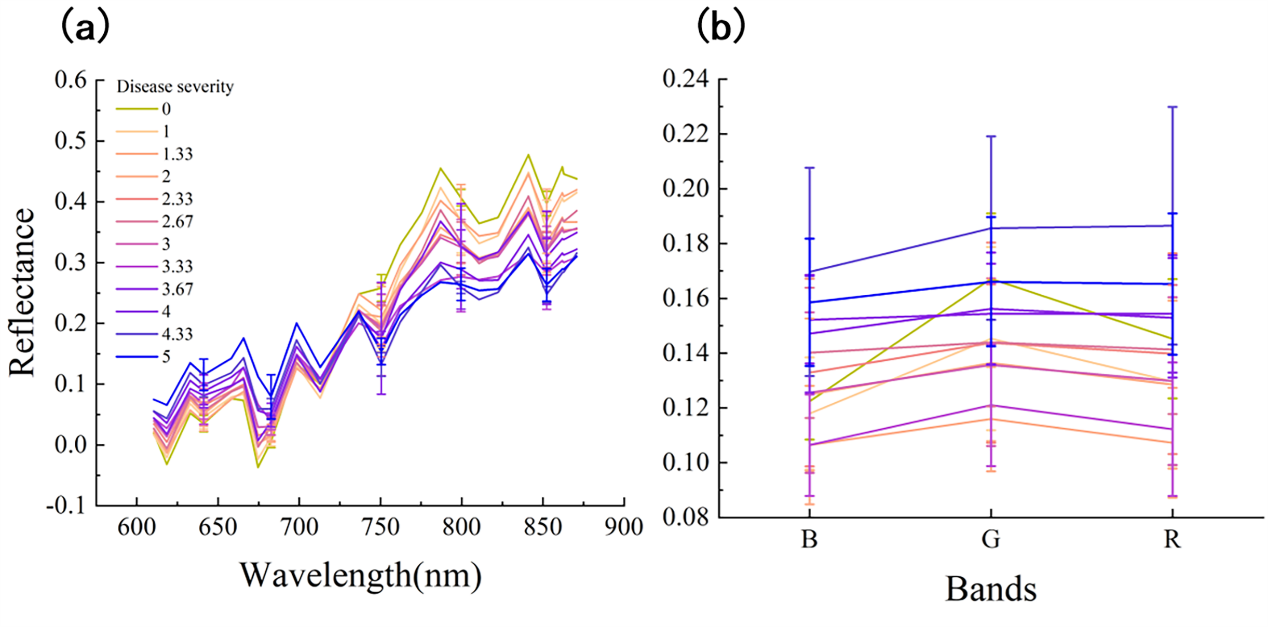** |
| --- |

**Figure S5. Averaged multispectral curves (A) and RGB spectral reflectance (B) of different disease severity in Experiment 1.** **The error bars represent the standard error of the mean.**

| **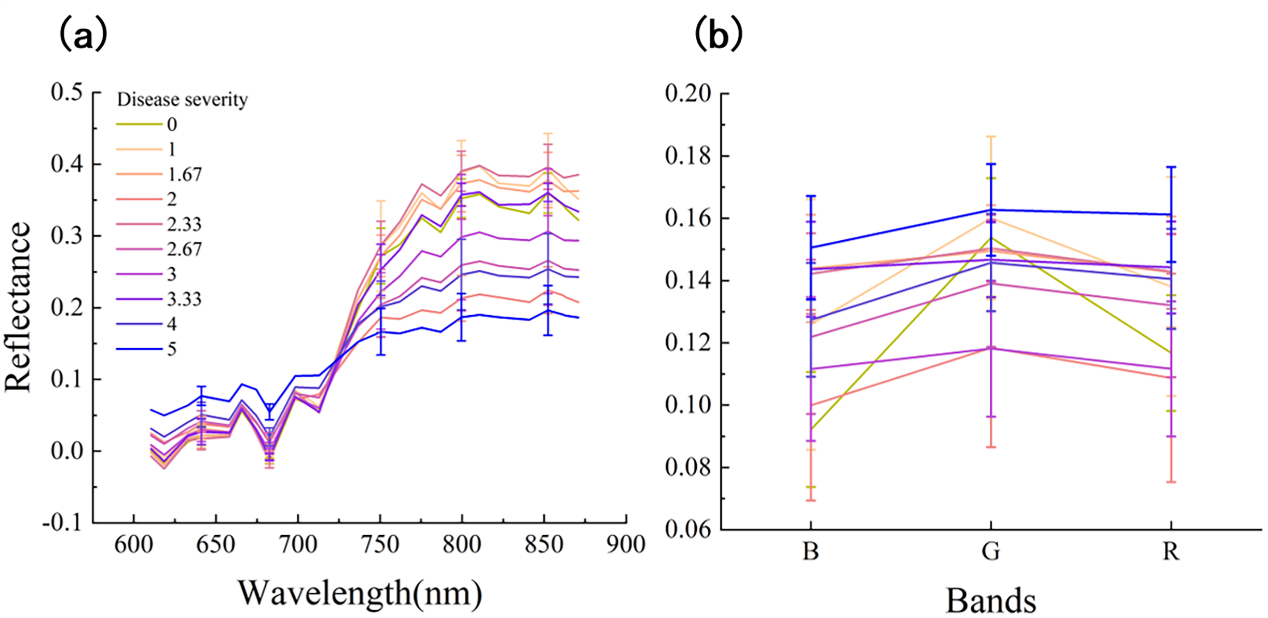** |
| --- |

**Figure S6. Averaged multispectral curves (A) and RGB spectral reflectance (B) of different disease severity in Experiment 2. The error bars represent the standard error of the mean.**

| **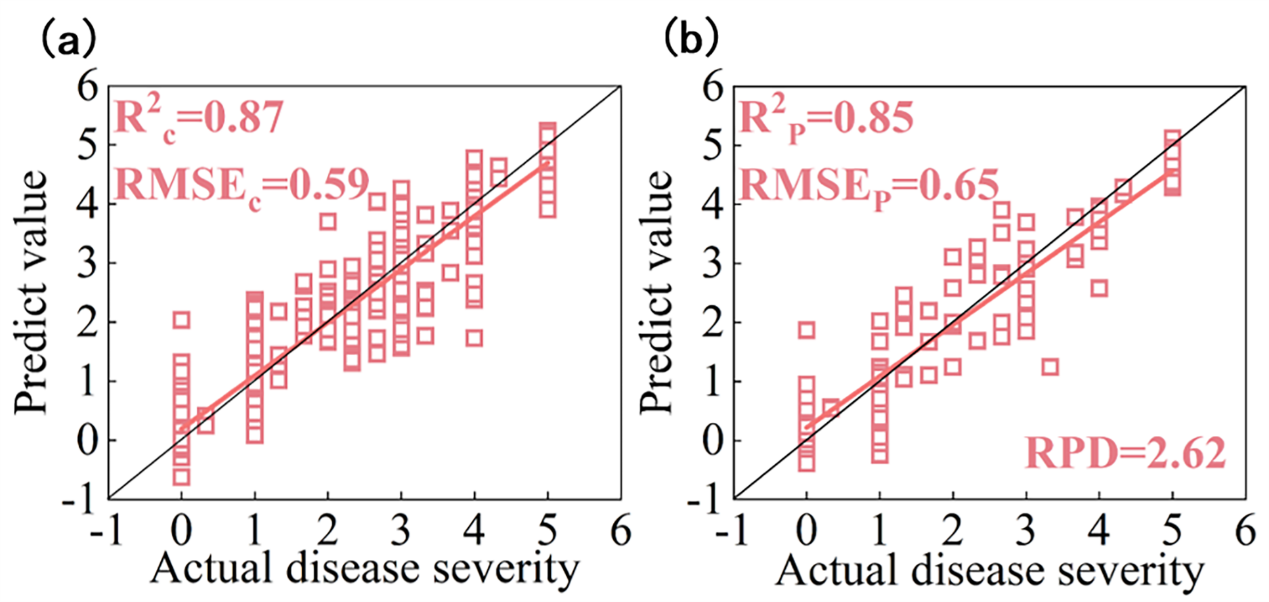** |
| --- |

**Figure S7. The results of calibration set (A) and prediction set (B) of the SVR model established for the evaluation of disease severity by using spectral data fused with AT data.**
